# An Account of an Improved Method of Treating the Puerperal Fever

**Published:** 1782

**Authors:** 


IV. *An account of an improved Method of treating the Puerperal Fever. By* Anthony Fothergill, M. D. *Member of the Royal College of Physicians, and Fellow of the Royal Society. Read* December 23, 1782.

TO convey early information of improvements in the cure of dangerous diseases, is a duty we owe to our brethren of the faculty, and to society. If medical facts were circulated with as much assiduity as political occurrences, it would tend not a little to the advancement of the art, and the preservation of life. Hence the utility of Medical Journals, which at stated periods communicate, in a small compass, the most important discoveries which are carrying on in different parts of the world. I was led into this train of thinking on reading a very important article of intelligence in the French *Journal de Medecine* of last month. The puerperal fever, which has too long triumphed over all the resources of nature, and too often baffled all the efforts of art, has at length, we are told, yielded to a very simple and easy method of cure happily discovered by M. Doulcet, one of the physicians to the Hotel Dieu at Paris.

 The

The fever, we are informed, commenced about the third day after delivery, often after the moſt natural labours, attended with ſevere pain, and tenſion of the abdomen; proſtration of ſtrength; the pulſe quick, ſmall, and contracted. The breaſts, inſtead of expanding, collapſed; and the ſecretion of milk was ſuſpended. The above ſymptoms, which characterized the diſeaſe, were obſerved in all the women who were attacked. Beſides theſe, ſhivering generally marked the onſet, accompanied with nauſea, vomiting, fœtid alvine dejections, &c. The *lochia* nevertheleſs continued to flow. A flattering remiſſion came on upon the ſecond day, but was ſoon followed by cold clammy ſweats, tremulous pulſe, delirium, and finally death, about the third or fourth day. On diſſection, were found in the cavity of the abdomen two or three pints of a milky or whey-like fluid, of a fœtid odour, and containing flakes of apparently coagulated milk, a large quantity of which firmly adhered to the ſurface of the inteſtines. The uterus was found in a natural ſtate. The diſeaſe being epidemic, and having proved uniformly fatal, in ſpite of all the various means of treatment which had been hitherto uſed at the Hotel Dieu, M. Doulcet marking the ſpontaneous efforts of vomiting, recomrecommended the following method on the firſt approach of the diſeaſe, viz. fifteen grains of ipecacoanha, in two doſes, at the interval of an hour and half. The emetic having operated, was determined downwards by an oily mixture, conſiſting of two ounces of oil of almonds, an ounce of ſyrup of marſhmallows, and two grains of kermes mineral. The ſame proceſs was repeated the next day, and purſued till the ſymptoms diſappeared. Linſeed tea, and other ſoft diluents, ſweetened with ſyrup of marſhmallows, for common drink. On the ſeventh or eighth day, a gentle cathartic of manna and purging ſalt. During ſome months that the diſeaſe raged in the Hoſpital, this method was crowned with ſucceſs in near 200 caſes, while five or ſix women, who refuſed to take the medicines, fell victims to their own obſtinacy. This remarkable ſucceſs being found to depend on combating the diſeaſe on its onſet, the midwife who ſuperintended the lying-in women, was charged to adminiſter the medicine at the very inſtant the firſt ſymptoms appeared, whether night or day. The author it ſeems is ſince dead; but his ſucceſsful method of cure being confirmed by the other phyſicians who attend the Hotel Dieu, and having received the approbation of the Royal Medical Society, who who were lately ordered by Government to enquire into its merit *, we can have no room to doubt the fact, altho' the theory may perhaps be controverted. It would, however, be premature either to condemn the one, or adopt the other, till both have undergone a candid examination by other practitioners. In the interim I beg leave to remark,

1st. That the suspension of the secretion of milk, which the learned author, and others, suppose to be the cause, ought perhaps rather to be deemed the consequence of the disease; since these suspensions often happen without producing it, and the disease is often removed without the secretion being restored, as appears even from their own account. The extravasated fluid and white coagulum, which they attribute to a real *metastasis* of milk, are, I apprehend, rather to be considered as morbid exsudations of serum and coagulable lymph, so often conspicuous on the inflamed surfaces of the different cavities, and which assume exactly the same appearances which they describe, where there could be no suspicion of the presence of milk.

* See the printed testimonial, attested by seven eminent Physicians, intitled, Rapport fait par ordre du Gouvernement sur la methode de M. Doulcet, &c.

2dly. From

2dly. From the phenomena both before and after death, the diſeaſe ſeems to have conſiſted of a combination of the inflammatory and putrid diatheſis, in which the latter was predominant, ſimilar to what we have frequently obſerved in this country. That the Pariſian malady was more highly malignant, and conſequently run its career with greater rapidity, will not appear wonderful to any one who has ever viſited that crowded receptacle of diſeaſe and contagion, the Hotel Dieu.

3dly. Having myſelf adminiſtered emetics of ipecacoanha at a more advanced period of the diſeaſe, as well as oily demulcents, though by no means with equal ſucceſs to that of M. Doulcet; and as many other practitioners, it is preſumed, have done the ſame, the preſent method cannot be deemed entirely new. The whole ſecret then ſeems to depend on the *early* exhibition of the medicine, which, by nipping the diſeaſe in the bud, prevents the ſubſequent abdominal congeſtion. Such an opportunity however but ſeldom occurs, as the phyſicians of this country are rarely called till the diſeaſe has made a conſiderable progreſs, and the viſcera have ſuſtained irreparable injury! In a matter ſo intereſting to humanity, I cannot omit expreſſing an earneſt wiſh, wiſh, that in future all practitioners in midwifery, eſpecially thoſe who attend lying-in hoſpitals, will, in imitation of M. Doulcet, attentively watch the firſt ſymptoms of this very alarming diſeaſe, and alſo, like him, engage the nurſes to do the ſame, that when the characteriſtic marks above-mentioned appear, not a moment may be loſt in having recourſe to his method of cure. If by a timely uſe thereof he was enabled to give an immediate check to the fever in ſuch a crowded hoſpital, is it not ſufficient encouragement for Britiſh practitioners to try its efficacy under more favourable circumſtances?

By the attentive obſervation of impartial judges a true eſtimate of this method will, it is hoped, be formed, its advantages and diſadvantages pointed out, and its due boundaries more clearly aſcertained. Though it cannot be ſuppoſed that ſo attentive a practitioner as M. Doulcet ſeems to have been, could have miſtaken the after pains for the firſt ſymptoms of the puerperal fever; yet the midwife might be thus impoſed on during his abſence, and induced to give the medicine prematurely. The after-pains, however, are very diſtinguiſhable; for inſtead of being conſtant, they have alternate acceſſions and remiſſions, are not attended with ſudden languor languor and febrile ſymptoms, and generally ceaſe before the third day, when the puerperal fever only uſually commences. Beſides, we are informed that thoſe women who took the remedy recovered, while thoſe who refuſed it died with all the ſtriking marks of the diſeaſe. But ſhould M. Doulcet's method be preſumed by ſome to have anſwered as a prophylactic, rather than as a curative, in ſome of the numerous caſes he mentions; yet this would rather increaſe than diminiſh its merit with thoſe who think it is beſt to err on the ſafeſt ſide, and that it is more deſirable to prevent than to cure diſeaſes. In other highly contagious fevers, an emetic of ipecacoanha given on the firſt ſigns of contagion being received into the body, has been known to put an immediate ſtop to the diſeaſe. Does ipecacoanha then, produce theſe happy effects, by exerting any ſpecific power over the contagious principle? Or by evacuating it in common with any other emetic? Or is it not rather, by its concuſſion, communicating a new ſpring to the ſyſtem by which the exhalent and abſorbent veſſels are excited, noxious matters expelled, and dangerous determinations to the vital organs prevented?

It muſt be left to future experience to determine, how far this method may be ſafe when the

 pain pain and tenfion denote a high degree of inflammation to prevail; and whether in any cafe the ipecacoanha poffeffes a fuperiority over antimonial or other emetics? Laftly, whether the increafed danger of fevers in the puerperal ftate does not depend on fome other principle befides that of increafed irritability of the fyftem? And why, in the beginning of this fever, thofe women whofe mind happens to be deeply impreffed with the idea of a fatal termination of the difeafe, fcarcely ever recover?

